# Burnout Syndrome among Emergency Physicians and Nurses in Abha and Khamis Mushait Cities, Aseer Region, Southwestern Saudi Arabia

**DOI:** 10.1155/2019/4515972

**Published:** 2019-02-18

**Authors:** Abdulghani M. Alqahtani, Nabil J. Awadalla, Safar A. Alsaleem, Awad S. Alsamghan, Mohammed Abadi Alsaleem

**Affiliations:** ^1^Family Medicine Specialist, Ministry of Health, Saudi Arabia; ^2^Associate Professor, Department of Family & Community Medicine, College of Medicine, King Khalid University, Abha, Saudi Arabia; ^3^Assistant Professor, Department of Family & Community Medicine, College of Medicine, King Khalid University, Abha, Saudi Arabia

## Abstract

**Objectives:**

To explore the magnitude and determinants of burnout among emergency physicians and nurses working at emergency departments of hospitals in Abha and Khamis Mushait cities.

**Subjects and Methods:**

A cross-sectional hospital-based study was conducted in emergency departments of hospitals in Abha and Khamis Mushait cities belonging to Ministry of Health. All physicians (n=95) and nurses (n=187) currently working at these sites were invited to participate in the study by filling a validated self-administered questionnaire including two main sections: personal and professional characteristics of physicians and nurses as well as Maslach burnout inventory (MBI) to assess the three components of the burnout syndrome: emotional exhaustion, depersonalization, and reduced personal accomplishment.

**Results:**

The study included 282 physicians and nurses. The age of more than half of them (54.3%) ranged between 31 and 35 years. Most of them (70.9%) were females. About two-thirds of the respondents (66.3%) were nurses while the remaining 33.7% were physicians. Majority of the emergency healthcare professionals (88.7%) had high emotional exhaustion. The prevalence of high depersonalization (cynicism) was 20.6% whereas that of low personal accomplishment was 41.1% among emergency healthcare professionals. The overall prevalence of burnout among healthcare professionals was 16.3%. Multivariate logistic regression analysis revealed that male healthcare professionals were at almost higher three-folded risk for developing burnout compared to females (aOR=2.76; 95% confidence interval (CI): 1.21-6.28, p=0.017)). Smokers were at higher significant risk for burnout compared to nonsmokers (aOR=15.37; 95% CI: 7.06-33.45, p<0.001). Healthcare professionals who reported a history of taking medications for sleep disorders expressed higher risk for burnout opposed to those with no history of sleep disorder medication (aOR=6.59; 95% CI: 2.08-20.81, p=0.001).

**Conclusion:**

A considerable proportion of physicians and nurses working at emergency departments of hospitals in Abha and Khamis Mushait cities had burnout syndrome, particularly high emotional exhaustion and low personal accomplishment.

## 1. Introduction

Working in emergency medicine field for both physicians and nurses is a stressful job as it involves dealing with a workplace circumstances, including overcrowding, shift work, critical decisions made with incomplete information, and death that have been associated with high burnout factors [1, 2].

Burn out syndrome is defined as “a syndrome of emotional exhaustion (EE), depersonalization (DP) (impersonal response towards patients), and reduced personal accomplishment (PA) among individuals who work with people” [3].

Clinical symptoms of burnout syndrome are nonspecific and include headaches, loss of energy, tiredness, lack of motivation, eating problems, irritability, insomnia, negative attitudes towards others, rigidity in relationships with other people, physical illness, and emotional instability [2, 4, 5].

Burnout syndrome imposes negative consequences on emergency (ER) physicians, their patients, and healthcare facilities as a result of disturbed mood of the medical staff, absenteeism, high rate of turnover, and patient dissatisfaction as a result of affection of the quality of care [6, 7]. In addition, conflicts with patients' companions may have a role in the development of burnout syndrome among them [8].

Emergency medicine workers (physicians and nurses) have been found to have a greater risk of burnout compared to other medical professionals [9].

Both job-related (years of practice, daily and weekly hours of work, professional development activities, nonclinical duties, and relationship with colleagues and higher staff) and non-job-related factors (demographics and lifestyle factors) are documented to be associated with burnout among health workers [10].

It has been suggested that sociodemographic factors have been linked to increasing job burnout rates, particularly age, gender, and marital status [11]. Additionally, professional, and organizational factors may influence the rate and level of burnout, particularly income, position, education, and work load [12]. Therefore, the sociodemographic and workplace-related factors were carefully elected to identify predictors for burnout among healthcare professionals [13].

There is a lack of evident consensus regarding the factors related to burnout, so, it is difficult for institutions to predict which members of their team are going to be burned out [2]. Clear understanding of the relationship between these factors and burnout could help responsible people in healthcare facilities emergency departments to take precautionary steps to build an effective workforce.

Although there are multiple burnout studies on emergency physicians from different parts of the world, we were unable to find any published studies from Aseer region. The aim of the study was to investigate the magnitude and determinants of burnout among emergency physicians and nurses working at emergency departments of hospitals in Abha and Khamis Mushait cities.

## 2. Methodology

### 2.1. Study Area

The study was conducted in Abha and Khamis Mushait cities located in the southwest of the kingdom of Saudi Arabia. It has an area of 851 square kilometres and an estimated population of 934,921 [14].

### 2.2. Study Design

A cross-sectional study design was used.

### 2.3. Study Settings

The study was carried out in emergency departments of hospitals belonging to Ministry of Health in Abha and khamis Mushait cities serving more than one hundred thousand patients yearly. These hospitals are 4 hospitals in Aseer region, namely, Aseer Central Hospital (600 beds), Maternity and Children Hospital in Abha city (200 beds), Khamis Mushait General Hospital (200 beds), and Maternity and Children Hospital in Khamis Mushait (200 beds).

### 2.4. Target Population/Sampling

All physicians (n=95) and nurses (n=187) currently working at Aseer Central Hospital, Maternity and Children Hospital in Abha city, Khamis Mushait General Hospital, and Maternity and Children Hospital in Khamis Mushait were invited to participate in the study by filling the study questionnaire.

### 2.5. Data Collection Tools

A validated self-administered questionnaire including two main sections was used for collecting data. The first part included questions regarding personal and professional characteristics of physicians and nurses (age, sex, marital status, nationality, job title, highest qualification, duration of work in the current facility, average duration of work (days/week and hours/day), on-call and average number of night shifts/month, smoking history, and medication history (against depression, anxiety and sleep disorders)). The second part included Maslach burnout inventory (MBI) English version [3]. The MBI is designed to assess the three components of the burnout syndrome: (emotional exhaustion, depersonalization, and reduced personal accomplishment). It consists of 22 items that measure burnout, and it is divided into three subscales: emotional exhaustion = (9 items), depersonalization (cynicism) = (5 items), and personal accomplishment = (8 items). For both the emotional exhaustion and depersonalization subscale, higher mean scores correspond to higher degree of experienced burnout. In contrast to these two subscales, lower mean scores on personal accomplishment subscale correspond to higher degree of experienced burnout. the items are written in the form of statements about personal feelings or attitudes and are answered in terms of the frequency with which the respondent experiences these feelings, on a 7-point Likert fully anchored (ranging from 0, “never”, to 6, “every day”). Each respondent's test form is scored by using a scoring key that contains directions for scoring each subscale. The scores for each subscale are considered separately and are not combined into a single total score; thus three scores are computed for each respondent. High emotional exhaustion was considered at score of 26 or more, high depersonalization was considered at score of 9 or more, and low personal accomplishment was considered at score of 33 or less.

Burnout was defined as high scores of emotional exhaustion and depersonalization and low score on personal accomplishment. It has become the gold standard for identifying burnout in the medical research literature which is found to be reliable and valid [3].

### 2.6. Pilot Study

A pilot study was conducted at one of the selected emergency departments on 10 physicians and 10 nurses to test wording of the questionnaire in order to avoid interobserver variation or bias. Minimal modification was done after pilot study. Data collection was totally performed by the researcher.

### 2.7. Administrative and Ethical Considerations

All the necessary official permissions were obtained before data collection. Prior to data collection, the investigator informed all potential participants regarding the objectives of the study. They were assured that no harm is ever expected to occur if they decide to participate in the study. They were also assured about the anonymity and full confidentiality of their responses. Their verbal consents to participate were requested.

### 2.8. Data Entry and Statistical Analysis

Statistical Package for Social Sciences (SPSS) software version 22.0 was used for data entry and analysis. Descriptive statistics (number, percentage for categorical variables and mean, standard deviation (SD), and range for continuous variables), and analytic statistics using Chi Square tests (*χ*^2^) to test for the association and/or the difference between two categorical variables were applied. Multivariate logistic regression analysis was applied to control the confounding effect. Burnout, based on MBI, was treated as dependent variable in multivariate logistic regression analysis. Significant job-related and non-job-related factors were treated as independent categorical variables. Each category of the independent variables was contrasted with the initial category (reference category). The adjusted odds ratio (OR) with 95% Confidence Interval (95% CI) were computed. P value less than 0.05 was considered statistically significant.

## 3. Results

### 3.1. Personal Characteristics of the Participants

**Figure 1:**
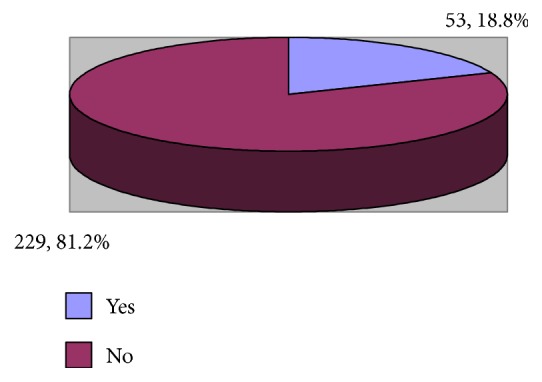
Smoking history of the emergency department physicians and nurses.

The study included 282 physicians and nurses. The age of more than half of them (54.3%) ranged between 31 and 35 years whereas that of 26.2% ranged between 25 and 30 years. Most of them (70.9%) were females. Slightly more than half of them (52.5%) were singles and 61.7% were non-Saudis. Regarding the highest qualification, 62.1% had diploma and only 4 (1.4%) had MD/equivalent (Table 1).  Figure 1 shows that smoking was reported by 18.8% of the emergency healthcare professionals.

**Figure 2:**
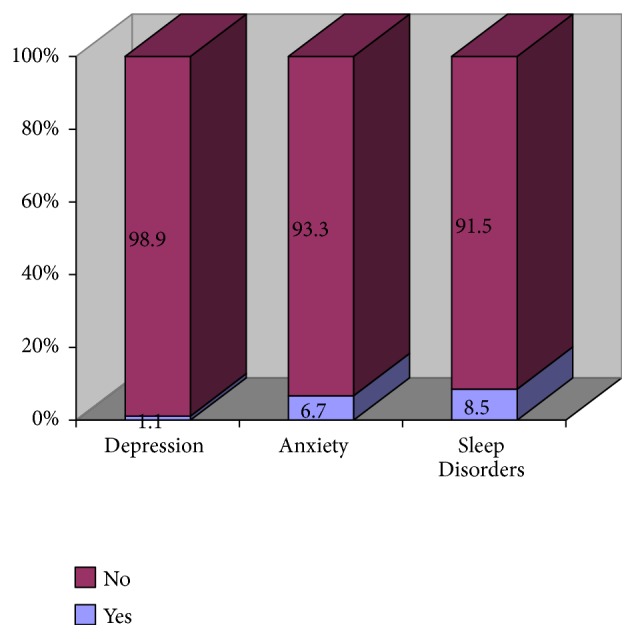
Medication history among emergency department physicians and nurses. Work-related characteristics of the participants.

**Figure 3:**
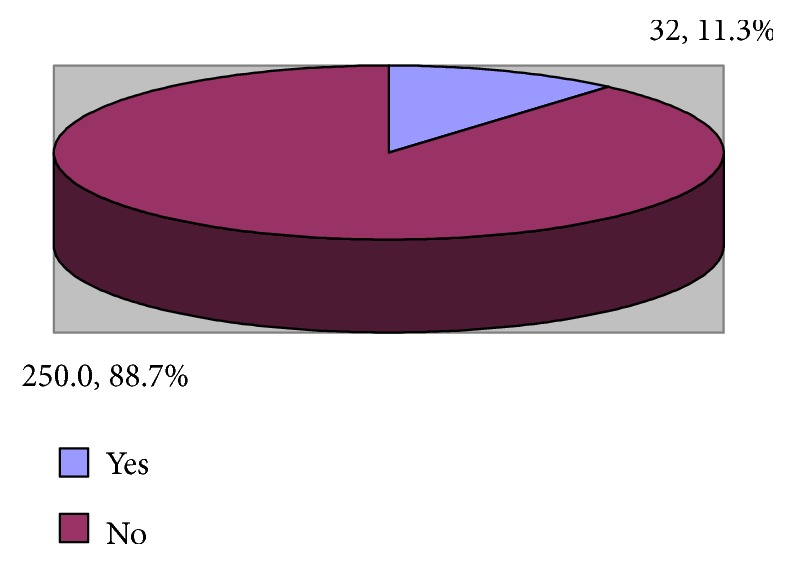
Prevalence of high emotional exhaustion among emergency department physicians and nurses.

As demonstrated from Figures 2 and 3 participants (1.1%) had a history of antidepressants intake whereas 19 participants (6.7%) had a history of intake of antianxiety medication, and 24 (8.5%) has a history of taking drugs for sleeping disorders.

In Table 2, about two-thirds of the respondents (66.3%) were nurses while the remaining 33.7% were physicians. Those working in Aseer Central Hospital (adults) represent 39.7% of them. Most of them had duration of work in the current facility ranged between one and three years whereas the overall duration ranged between one and ten years with a mean of 2.7 and SD of 1.6 years. Half of them work either 5 or 6 days/week and all work 8 hours per day. Approximately two-thirds (61%) of the emergency healthcare professionals had 6 weeks as annual vacation. On-call duties were reported by only 18.4% of them. Night shifts ranged on the average between 6 and 9/month among 66% of the participants and overall ranged between one and 12 with a median of 8 shifts.

### 3.2. High Emotional Exhaustion

It is realized from Figure 3 that the majority of the emergency healthcare professionals (88.7%) had high emotional exhaustion.

From Table 3, it is seen that there was a statistically significant association between age of the participants and high emotional exhaustion as 94.8% of those aged between 31 and 35 years compared to 78.4% of those aged between 25 and 30 years that had high EE, p=0.002. Female healthcare professionals had higher EE compared to males (93% versus 78%), p<0.001. Regarding nationality, non-Saudis had significantly higher EE compared to Saudis (93.7% versus 80.6%), p=0.001. Lower qualified healthcare professionals (Diploma holders) had higher rate of high EE compared to Master holders (92% versus 52.4%), p<0.001. Nurses had higher EE compared to physicians (92.5% versus 81.1%), p=0.004. All healthcare professionals working in Abha Maternity/Pediatric Hospital (Pediatric ER) compared to 79.5% of those working in Aseer Central Hospital (adults) expressed high EE, p=0.004. Majority of healthcare professionals who had 6 weeks annual vacation (93%) compared to 80% of those who had 5 weeks of annual vacation had high EE, p=0.015. Healthcare professionals who had no on-call duties expressed higher EE than those who had on-call duties (90.4% versus 80.8%), p=0.047. All participants who had medications for sleep disturbance compared to 87.6% of those who had no such history expressed high EE, p=0.049. Other studied factors (marital status, duration of work in the current facility, average number of working days/ week, average number of night shifts/month, smoking history, history of antidepressants, and antianxiety medication) were not significantly associated with high EE.

### 3.3. High Depersonalization (Cynicism)

**Figure 4:**
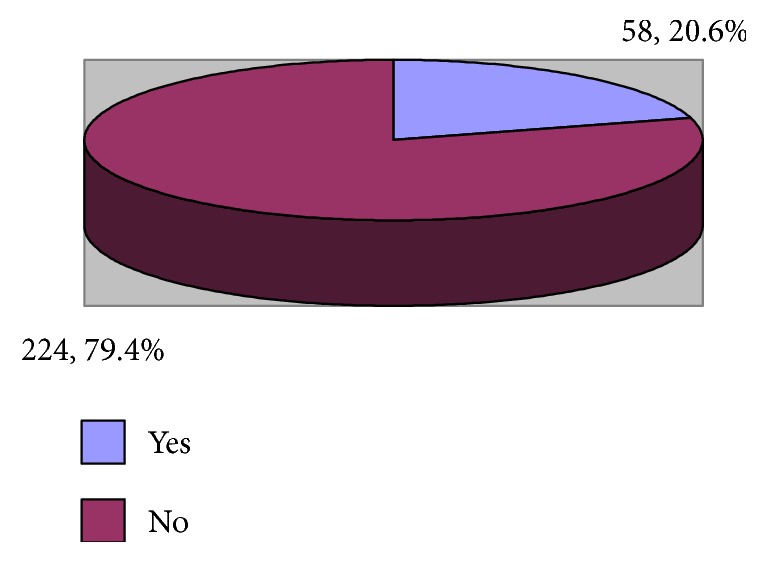
Prevalence of high depersonalization (cynicism) among emergency department physicians and nurses.

It is evident from Figure 4 that 20.6% of the emergency healthcare professionals had high depersonalization (cynicism).

Table 4 illustrates that there was a statistically significant association between place of work of the participants and high DP as 37.1% of those working in Khamis Mushait general hospital compared to 6.1% of those working in Khamis Mushait Maternity and Pediatric Hospital (Maternity ER) who had high DP, p=0.037. Male healthcare professionals had higher DP compared to females (30.5% versus 16.5%), p=0.008. Healthcare professionals who had on-call duties expressed higher DP than those who had no on-call duties (32.7% versus 17.8%), p=0.017. Healthcare professionals who had medications for sleep disturbance showed higher rate of high DP compared to those who had no such history (41.7% versus 18.6%), p=0.008. Smoker healthcare professionals expressed higher significant rate of high DP compared to nonsmokers (60.4% versus 11.4), p<0.001. Other studied factors (age, nationality, marital status, qualification, job title, duration of work in the current facility, average number of working days/week, duration of annual vacation, average number of night shifts/month, history of antidepressants, and antianxiety medication) were not significantly associated with high DP.

### 3.4. Low Personal Accomplishment

**Figure 5:**
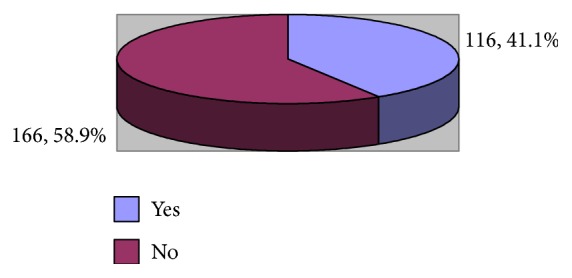
Prevalence of low personal accomplishment among emergency department physicians and nurses.

As shown from Figure 5, the prevalence of low personal accomplishment among emergency healthcare professionals was 41.1%.

From Table 5, it is shown that there was a statistically significant association between age of the participants and low personal accomplishment as 50% of those aged between 25 and 30 years compared to 22.7% of those aged between 36 and 40 years who had low PA, p=0.037. Single healthcare professionals had higher low PA compared to those who are married (51.4% versus 29.2%), p<0.001. Regarding nationality, Saudis had significantly higher low PA compared to non-Saudis (48.1% versus 36.8%). However, the difference did not reach a statistically significant level. Lower qualified healthcare professionals (diploma holders) had higher rate of low PA compared to Master holders (48% versus 23.8%), p-0.024. Nurses had higher low PA compared to physicians (48.1% versus 27.4%), p=0.001. Almost half (51%) of healthcare professionals who had 4 weeks of annual vacation compared to 20% of those who had 5 weeks of annual vacation had low PA, p=0.026. Healthcare professionals had >9 night shifts/month expressed higher significant rate of low PA compared to those who had ≤5 night shifts/moth (52.4% versus 24.2%), p=0.027. Smoker healthcare professionals expressed higher significant rate of low PA compared to nonsmokers (73.6% versus 33.6), p<0.001. Majority of participants who had medications for anxiety (94.7%) compared to 37.3% of those who had no such history expressed high rate of low PA, p<0.001. Also, majority of participants who had medications for sleep disorder (91.7%) compared to 36.4% of those who had no such history expressed high rate of low PA, p<0.001. Other studied factors (gender, place of work, duration of work in the current facility, average number of working days/week, on-call duties, and history of antidepressants) were not significantly associated with lowPA.

### 3.5. Burnout

**Figure 6:**
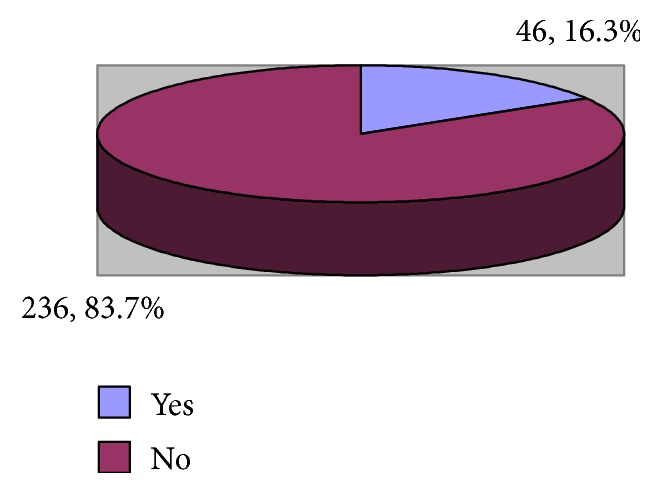
Prevalence of burnout among emergency department physicians and nurses.

Figure 6 shows that the prevalence of burnout among healthcare professionals was 16.3%. From Table 6, it is shown that male healthcare professionals had higher significant rate of burnout compared to females (25.6% versus 12.5%), p=0.007. Healthcare professionals who had on-call duties expressed higher significant rate of burnout compared to those without on-call duties (26.9% versus 13.9%), p=0.022. Smoker healthcare professionals expressed higher significant rate of burnout compared to nonsmokers (56.6% versus 7), p<0.001. Almost one-third of participants who had medications for anxiety (36.8%) compared to 14.8% of those who had no such history expressed high rate of burnout, p=0.012. Also, 41.7% of participants who had medications for sleep disorder compared to 14% of those who had no such history expressed high rate of burnout, p<0.001. Other studied factors (age, marital status, nationality, highest qualification, job title, place of work, duration of work in the current facility, average number of working days/week, duration of annual vacation, average number of night shifts, and history of antidepressants) were not significantly associated with burnout.

### 3.6. Predictors of Burnout

Multivariate logistic regression analysis in Table 7 revealed that male healthcare professionals were at almost higher three-folded risk for developing burnout compared to females (adjusted odds ratio (aOR)=2.76; 95% confidence interval (CI): 1.21-6.28, p=0.017). Smokers were at higher significant risk for burnout compared to nonsmokers (aOR=15.37; 95% CI: 7.06-33.45, p<0.001). Healthcare professionals who reported a history of taking medications for sleep disorders expressed higher risk for burnout opposed to those with no history of sleep disorder medication (aOR=6.59; 95% CI: 2.08-20.81, p=0.001. On-call duties and intake of antianxiety medications were not significantly associated with burnout after controlling for confounders.

## 4. Discussion

Emergency rooms are the most stressful departments of hospitals and as a result of inadequate physical working environment (overcrowding, rotational work schedules, conflict with patients/relatives, and unsafe workplace) and emotional circumstances (severity of cases, critical care decisions), their staff are at higher risk of burnout [8]. The aim of this study was to investigate the magnitude and determinants of burnout among emergency physicians and nurses working at emergency departments of hospitals in Abha and Khamis Mushait cities, Saudi Arabia

In the current study, a rate of burnout of 16.3% has been reported among ER healthcare professionals in Abha and Khamis Mushait cities, Saudi Arabia (18.9% among physicians and 15% among nurses). The results of other studies are controversial, with some studies reported alarming high rates of burnout among emergency staff, while others reported much lower rates. Higher figures than ours have been reported in other Saudi studies carried out among emergency physicians in Makkah, Riyadh, and Jeddah cities (48.7%) [15]. In Brazil (2009), a rate of 8.2% has been reported among female nursing staff from an ER department of a university hospital and a greater percentage (54.1%) of the nurses was at a higher risk for burnout [16].

In the current study, high EE was reported among 88.7% of the healthcare emergency staff; being significantly higher in nurses (92.5%) than physicians (81.1%), high depersonalization was reported among 20.6% of them, with no difference between physicians and nurses, whereas low PA was reported among 41.1% of the emergency staff, being higher significantly in nurses (48.1%) than physicians (27.4%). Nursing staff are most likely to develop higher EE due to their job nature, with several duties, greater time spent with patients and relatives, and the emotional demands of their work [17].

Different figures of burnout domains have been reported from different studies. In Riyadh (Saudi Arabia), the prevalence of high EE score was 40%, high DP score was 40%, and low PA score was 32% among emergency physicians working at emergency departments [18]. In Egypt [19], 46.9% of emergency staff had high EE, 97.7% showed low PA, and 14.4% expressed high depersonalization. In Turkey [8], 44.7% of emergency department staff showed high EE, 33.2% had high depersonalization, and 28% showed low PA.

In accordance with Abdo et al. [19] and Gosseries et al. [20], the present study revealed that nurses experienced high EE compared to physicians. However, in Spain, the prevalence of emotional exhaustion and low personal accomplishment were higher among doctors than nurses [21]. In a recent Turkish study (2016), EE and DP scores were high across all occupational groups (physicians, nurses, and medical technicians), while scores on PA were low. There was a statistically significant difference between nurses and medical technicians for EE and between physicians and both nurses and medical technicians for PA, while no group differences were found for DP [22].

The difference in the prevalence of burnout and its domains across studies can be attributed to culture variation, the nature of the healthcare system, attitude of patients and the healthcare staff at emergency situations, and the differences in the study design and assessment tools utilized in the various studies of burnout [23].

Logistic regression analysis in the current study revealed that male gender, having history of smoking and intake of medications for sleep disorders, was significantly associated with higher risk of burnout among emergency staff. Other studies reported that females were at higher risk of burnout compared to males in ER departments [15, 24]. This could be attributed to biological factors. However, males are more likely to experience work conflicts and responsibilities.

Younger emergency staff members were more likely to have low personal accomplishment than older members in the present study. This could be due to the fact that they face greater pressure to increase skills and knowledge through practice. In addition, their lack of enough experience may expose them to increased working hours. The same had been observed in a study carried out in Egypt among emergency physicians [19].

In Egypt, age, years of experience, frequency of exposure to violence at work, work burden, supervision, and work activities were significant determinants of burnout among emergency medical staff [19]. Arora et al. (2013) reported in their review that both work-related (hours of work, years of practice, professional development activities, nonclinical duties, etc.) and non-work-related factors (age, sex, lifestyle factors, etc.) are associated with burnout [25]. In Canada (2015), Howlett et al. concluded that task-oriented coping was associated with decreased risk of burnout, while emotion-oriented coping was associated with increased risk of burnout [26]. In Brazil (2009), factors associated with burnout among female emergency nursing staff were lack of knowledge and motivation for professional development [16]. Alaslani et al. (2016) found that younger (≤25 years), female, non-Saudi, low experienced, working more hours, and on-call emergency physicians working at Makkah, Riyadh, and Jeddah Saudi cities were more likely to express high emotional exhaustion compared to others [15]. In Turkey (2016), age, gender, and economic well-being were all significant predictors for burnout among emergency staff [22]. In the present study, place of work was significantly associated with high EE and DP. Further detailed study exploring the work nature in these places is warranted to explain this association more clearly.

Several studies showed a significant link between work hours and burnout [15, 27, 28]. However, others agree with the present study and failed to show such association [29].

The most important limitation of this study is the cross-sectional design which proves only association and not causality. However, conduction of the study in different general hospitals in various areas of the Region increases the generalizability of results. The results of this study will help in exploring the psychosocial work environment of emergency staff in Abha and Khamis Mushait cities, Saudi Arabia.

## 5. Conclusion

A considerable proportion of physicians and nurses working at emergency departments of main hospitals in Abha and Khamis Mushait cities had burnout syndrome, particularly high emotional exhaustion, and low personal accomplishment. Males, smokers, and those on medications for sleep disorder were more likely than others to suffer from burnout. Work-related characteristics were not associated with burnout syndrome.

## 6. Recommendations

Based on the current study, we can recommend the following:

1. Development of periodic (annually/with recruitment) screening system to recognize early signs of impairment and distress of ER staff by using MBI questionnaire.
2. Organizing lessons for ER staff on stress-management techniques that teach them how to cope better with stressful situations.
3. Organizing practical lessons for ER staff on time management, understanding personal values, motives, and goals.
4. Recruitment of ER staff in the appropriate places according to their qualifications and interest.
5. Further longitudinal cohort study is needed to identify the causation of burnout among ER staff in Abha and Khamis Mushait cities.

## Figures and Tables

**Table 1:** Personal characteristics of the emergency department physicians and nurses (n=282).

Personal characteristics	Frequency	Percentage
*Age (years)*		
25-30	74	26.2
31-35	153	54.3
36-40	44	15.6
>40	11	3.9

*Sex*		
Male	82	29.1
Female	200	70.9

*Marital status*		
Single	148	52.5
Married	134	47.5

*Nationality*		
Saudi	108	38.3
Non-Saudi	174	61.7

*Highest qualification*		
Diploma	175	62.1
Bachelor	82	29.1
Master	21	7.4
MD/equivalent	4	1.4

**Table 2:** Work-related characteristics of the emergency department healthcare professionals.

Work-related characteristics	Frequency	Percentage
*Job title*		
Physician	95	33.7
Nurse	187	66.3

*Place of work*		
KhamisMushait general H	35	12.4
Abha Mat/Ped H (Ped ER)	30	10.6
Abha Mat/Ped H (Mat ER)	34	12.1
K. M Mat/Ped H (Ped ER)	38	13.5
K. M Mat/Ped H (Mat ER)	33	11.7
Aseer Central hospital (adults)	112	39.7

*Duration of work in emergencydepartment (years)*		
1-3	230	81.6
>3	52	18.4
Range	1-10	
Mean±SD	2.7±1.6	

*Average number of working days/ week*		
Five	141	50.0
Six	141	50.0

*Average number of working hours/day*		
8	282	100

*Duration of annual vacation in weeks*		
4	100	35.5
5	10	3.5
6	172	61.0

On-call duties		
Yes	52	18.4
No	230	81.6

*Average number of night shifts/month*		
≤5	33	11.7
6-9	186	66.0
>9	63	22.3

*Range*	1-12	
*Median*	8	

**Table 3:** Factors associated with high emotional exhaustion among emergency healthcare professionals.

	High emotional exhaustion		p-value*∗*
	No	Yes	
	N=32	M=250	
	N (%)	N (%)	
*Age (years)*			
25-30 (n=74)	16 (21.6)	58 (78.4)	
31-35 (n=153)	8 (5.2)	145 (94.8)	
36-40 (n=44)	7 (15.9)	37 (84.1)	
>40 (n=11)	1 (9.1)	10 (90.5)	0.002

*Sex*			
Male (n=82)	18 (22.0)	64 (78.0)	
Female (n=200)	14 (7.0)	186 (93.0)	<0.001

*Marital status*			
Single (n=148)	18 (12.2)	130 (87.8)	
Married (n=134)	14 (10.4)	120 (89.6)	0.650

*Nationality*			
Saudi (n=108)	21 (19.4)	87 (80.6)	
Non-Saudi (n=174)	11 (6.3)	163 (93.7)	0.001

*Highest qualification*			
Diploma (n=175)	14 (8.0)	161 (92.0)	
Bachelor (n=82)	7 (8.5)	75 (91.5)	
Master (n=21)	10 (47.6)	11 (52.4)	
MD/equivalent (n=4)	1 (25.0)	3 (75.0)	<0.001

*Job title*			
Physician (n=95)	18 (18.9)	77 (81.1)	
Nurse (n=187)	14 (7.5)	173 (92.5)	0.004

*Place of work*			
KhamisMushait (K. M.) general H (n=35)	3 (8.6)	32 (91.4)	
Abha Mat/Ped H (Ped ER) (n=30)	0 (0.0)	30 (100)	
Abha Mat/Ped H (Mat ER) (n=34)	3 (8.8)	31 (91.2)	
K. M.Maternity and Pediatric H (Ped ER) (n=38)	2 (5.3)	36 (94.7)	
K. M Maternity and pediatric H (Mat ER) (n=33)	1 (3.0)	32 (97.0)	
Aseer Central hospital (adults) (n=112)	23 (20.5)	89 (79.5)	0.004

*Duration of work in the current facility (years)*			
1-3 (n=230)	25 (10.9)	205 (89.1)	
>3 (n=52)	7 (13.5)	45 (86.5)	0.595

*Average number of working days/ week*			
Five (n=141)	20 (14.2)	121 (85.6)	
Six (n=141)	12 (8.5)	129 (91.5)	0.133

*Duration of annual vacation in weeks*			
4 (n=100)	18 (18.0)	82 (82.0)	
5 (n=10)	2 (20.0)	8 (80.0)	
6 (n=172)	12 (70.0)	160 (93.0)	0.015

*On-call duties*			
Yes (n=52)	10 (19.2)	42 (80.8)	
No (n=230)	22 (9.6)	208 (90.4)	0.047

*Average number of night shifts/month*			
≤5 (n=33)	3 (9.1)	30 (90.9)	
6-9 (n=186)	22 (11.8)	164 (88.2)	
>9 (n=63)	7 (11.1)	56 (88.9)	0.899

*Smoking history*			
Yes (n=53)	4 (7.5)	49 (92.5)	
No (n=229)	28 (12.2)	201 (87.8)	0.240*∗∗*

*Anti-depressant medications*			
Yes (n=3)	0 (0.0)	3 (100)	
No (n=279)	32 (11.5)	247 (88.5)	0.696*∗∗*

*Medications for anxiety*			
Yes (n=19)	0 (0.0)	19 (100)	
No (n=263)	32 (12.2)	231 (87.8)	0.093*∗∗*

*Medications for sleep disorders*			
Yes (n=24)	0 (0.0)	24 (100)	
No (n=258)	32 (12.4)	226 (87.6)	0.049*∗∗*

*∗*Chi-square test; *∗∗* Fischer exact test.

**Table 4:** Factors associated with high depersonalization (cynicism) among emergency healthcare professionals.

	High depersonalization		p-value*∗*
	No	Yes	
	N=224	M=58	
	N (%)	N (%)	
*Age (years)*			
25-30 (n=74)	59 (79.7)	15 (20.3)	
31-35 (n=153)	122 (79.7)	31 (20.3)	
36-40 (n=44)	36 (81.8)	8 (18.2)	
>40 (n=11)	7 (63.6)	4 (36.4)	0.605

*Sex*			
Male (n=82)	57 (69.5)	25 (30.5)	
Female (n=200)	167 (83.5)	33 (16.5)	0.008

*Marital status*			
Single (n=148)	114 (77.0)	34 (23.0)	
Married (n=134)	110 (82.1)	24 (17.9)	0.294

*Nationality*			
Saudi (n=108)	84 (77.8)	24 (22.2)	
Non-Saudi (n=174)	140 (80.5)	34 (19.5)	0.588

*Highest qualification*			
Diploma (n=175)	141 (80.6)	34 (19.4)	
Bachelor (n=82)	63 (76.8)	19 (23.2)	
Master (n=21)	17 (81.0)	4 (19.0)	
MD/equivalent (n=4)	3 (75.0)	1 (25.0)	0.906

*Job title*			
Physician (n=95)	72 (75.8)	23 (24.2)	
Nurse (n=187)	152 (81.3)	35 (18.7)	0.281

*Place of work*			
Khamis Mushait (K. M.) general H (n=35)	22 (62.9)	13 (37.1)	
Abha Mat/Ped H (Ped ER) (n=30)	25 (83.3)	5 (16.7)	
Abha Mat/Ped H (Mat ER) (n=34)	26 (76.5)	8 (23.5)	
K. M. Maternity and Pediatric H (Ped ER) (n=38)	28 (73.7)	10 (26.3)	
K. M Maternity and pediatric H (Mat ER) (n=33)	31 (93.9)	2 (6.1)	
Aseer Central hospital (adults) (n=112)	92 (82.1)	20 (17.9)	0.037

*Duration of work in the current facility (years)*			
1-3 (n=230)	184 (80.0)	46 (20.0)	
>3 (n=52)	40 (76.9)	12 (23.1)	0.620

*Average number of working days/ week*			
Five (n=141)	110 (78.0)	31 (22.0)	
Six (n=141)	114 (80.9)	27 (19.1)	0.556

*Duration of annual vacation in weeks*			
4 (n=100)	78 (78.0)	22 (22.0)	
5 (n=10)	7 (70.0)	3 (30.0)	
6 (n=172)	139 (80.8)	33 (19.2)	0.647

*On-call duties*			
Yes (n=52)	35 (67.3)	17 (32.7)	
No (n=230)	189 (82.2)	41 (17.8)	0.017

*Average number of night shifts/month*			
≤5 (n=33)	28 (84.8)	5 (15.2)	
6-9 (n=186)	147 (79.0)	39 (21.0)	
>9 (n=63)	49 (77.8)	14 (22.2)	0.699

*Smoking history*			
Yes (n=53)	21 (39.6)	32 (60.4)	
No (n=229)	203 (88.6)	26 (11.4)	<0.001

*Anti-depressant medications*			
Yes (n=3)	1 (33.3)	2 (66.7)	
No (n=279)	223 (79.9)	56 (20.1)	0.108*∗∗*

*Medications for anxiety*			
Yes (n=19)	12 (63.2)	7 (36.8)	
No (n=263)	212 (80.6)	51 (19.4)	0.069

*Medications for sleep disorders*			
Yes (n=24)	14 (58.3)	10 (41.7)	
No (n=258)	210 (81.4)	48 (18.6)	0.008

*∗*Chi-square test; *∗∗* Fischer exact test.

**Table 5:** Factors associated with low personal accomplishment among emergency healthcare professionals.

	Low personal accomplishment		p-value*∗*
	No	Yes	
	N=166	M=116	
	N (%)	N (%)	
*Age (years)*			
25-30 (n=74)	37 (50.0)	37 (50.0)	
31-35 (n=153)	89 (58.2)	64 (41.6)	
36-40 (n=44)	34 (77.3)	10 (22.7)	
>40 (n=11)	6 (54.5)	5 (45.5)	0.037

*Sex*			
Male (n=82)	54 (65.9)	28 (34.1)	
Female (n=200)	112 (56.0)	88 (44.0)	0.127

*Marital status*			
Single (n=148)	72 (48.6)	76 (51.4)	
Married (n=134)	94 (70.1)	40 (29.2)	<0.001

*Nationality*			
Saudi (n=108)	56 (51.9)	52 (48.1)	
Non-Saudi (n=174)	110 (63.2)	64 (36.8)	0.059

*Highest qualification*			
Diploma (n=175)	91 (52.0)	84 (48.0)	
Bachelor (n=82)	56 (68.3)	26 (31.7)	
Master (n=21)	16 (76.2)	5 (23.8)	
MD/equivalent (n=4)	3 (75.0)	1 (25.0)	0.024

*Job title*			
Physician (n=95)	69 (72.6)	26 (27.4)	
Nurse (n=187)	97 (51.9)	90 (48.1)	0.001

*Place of work*			
Khamis Mushait (K. M.) general H (n=35)	15 (42.9)	20 (57.1)	
Abha Mat/Ped H (Ped ER) (n=30)	19 (63.3)	11 (36.7)	
Abha Mat/Ped H (Mat ER) (n=34)	17 (50.0)	17 (50.0)	
K. M. Maternity and Pediatric H (Ped ER) (n=38)	23 (60.5)	15 (39.5)	
K. M Maternity and pediatric H (Mat ER) (n=33)	19 (57.6)	14 (42.4)	
Aseer Central hospital (adults) (n=112)	73 (65.2)	39 (34.8)	0.223

*Duration of work in the current facility (years)*			
1-3 (n=230)	130 (56.5)	100 (43.5)	
>3 (n=52)	36 (59.2)	16 (30.8)	0.093

*Average number of working days/ week*			
Five (n=141)	87 (61.7)	54 (38.3)	
Six (n=141)	79 (56.0)	62 (44.0)	0.333

*Duration of annual vacation in weeks*			
4 (n=100)	49 (49.0)	51 (51.0)	
5 (n=10)	8 (80.0)	2 (20.0)	
6 (n=172)	109 (63.4)	63 (36.6)	0.026

*On-call duties*			
Yes (n=52)	30 (57.7)	22 (42.3)	
No (n=230)	136 (59.1)	94 (40.9)	0.849

*Average number of night shifts/month*			
≤5 (n=33)	25 (75.8)	8 (24.2)	
6-9 (n=186)	111 (59.7)	75 (40.3)	
>9 (n=63)	30 (47.6)	33 (52.4)	0.027

*Smoking history*			
Yes (n=53)	14 (26.4)	39 (73.6)	
No (n=229)	152 (66.4)	77 (33.6)	<0.001

*Anti-depressant medications*			
Yes (n=3)	0 (0.0)	3 (100)	
No (n=279)	166 (59.5)	113 (40.5)	0.069*∗∗*

*Medications for anxiety*			
Yes (n=19)	1 (5.3)	18 (94.7)	
No (n=263)	165 (62.7)	98 (37.3)	<0.001*∗∗*

*Medications for sleep disorders*			
Yes (n=24)	2 (8.3)	22 (91.7)	
No (n=258)	164 (63.6)	94 (36.4)	<0.001*∗∗*

*∗*Chi-square test; *∗∗* Fischer exact test.

**Table 6:** Factors associated with burnout among emergency healthcare professionals.

	Burnout		p-value*∗*
	No	Yes	
	N=236	M=46	
	N (%)	N (%)	
*Age (years)*			
25-30 (n=74)	60 (81.1)	14 (18.9)	
31-35 (n=153)	129 (84.3)	24 (15.7)	
36-40 (n=44)	40 (90.9)	4 (9.1)	
>40 (n=11)	7 (63.6)	4 (36.4)	0.149

*Sex*			
Male (n=82)	61 (74.4)	21 (25.6)	
Female (n=200)	175 (87.5)	25 (12.5)	0.007

*Marital status*			
Single (n=148)	120 (81.1)	28 (18.9)	
Married (n=134)	116 (86.6)	18 (13.4)	0.213

*Nationality*			
Saudi (n=108)	86 (79.6)	22 (20.4)	
Non-Saudi (n=174)	150 (86.2)	24 (13.8)	0.146

*Highest qualification*			
Diploma (n=175)	148 (84.6)	27 (15.4)	
Bachelor (n=82)	67 (81.7)	15 (18.3)	
Master (n=21)	18 (85.7)	3 (14.3)	
MD/equivalent (n=4)	3 (75.0)	1 (25.0)	0.892

*Job title*			
Physician (n=95)	77 (81.1)	18 (18.9)	
Nurse (n=187)	159 (85.0)	28 (15.0)	0.393

*Place of work*			
Khamis Mushait (K. M.) general H (n=35)	25 (71.4)	10 (28.6)	
Abha Mat/Ped H (Ped ER) (n=30)	26 (86.7)	4 (13.3)	
Abha Mat/Ped H (Mat ER) (n=34)	27 (79.4)	7 (20.6)	
K. M. Maternity and Pediatric H (Ped ER) (n=38)	32 (84.2)	6 (15.8)	
K. M Maternity and pediatric H (Mat ER) (n=33)	31 (93.9)	2 (6.1)	
Aseer Central hospital (adults) (n=112)	95 (84.8)	17 (15.2)	0.209

*Duration of work in the current facility (years)*			
1-3 (n=230)	196 (85.2)	34 (14.8)	
>3 (n=52)	40 (76.9)	12 (23.1)	0.144

*Average number of working days/ week*			
Five (n=141)	116 (82.3)	25 (17.7)	
Six (n=141)	120 (85.1)	21 (14.9)	0.519

*Duration of annual vacation in weeks*			
4 (n=100)	79 (79.0)	21 (21.0)	
5 (n=10)	8 (80.0)	2 (20.0)	
6 (n=172)	149 (86.6)	23 (13.4)	0.247

*On-call duties*			
Yes (n=52)	38 (73.1)	14 (26.9)	
No (n=230)	198 (86.1)	32 (13.9)	0.022

*Average number of night shifts/month*			
≤5 (n=33)	29 (87.9)	4 (12.1)	
6-9 (n=186)	155 (83.3)	31 (16.7)	
>9 (n=63)	52 (82.5)	11 (17.5)	0.778

*Smoking history*			
Yes (n=53)	23 (43.4)	30 (56.6)	
No (n=229)	213 (93.0)	16 (7.0)	<0.001

*Anti-depressant medications*			
Yes (n=3)	1 (33.3)	2 (66.7)	
No (n=279)	235 (84.2)	44 (15.9)	0.070*∗∗*

*Medications for anxiety*			
Yes (n=19)	12 (63.2)	7 (36.8)	
No (n=263)	224 (85.2)	39 (14.8)	0.012

*Medications for sleep disorders*			
Yes (n=24)	14 (58.3)	10 (41.7)	
No (n=258)	222 (86.0)	36 (14.0)	<0.001

*∗*Chi-square test; *∗∗* Fischer exact test.

**Table 7:** Predictors of burnout among emergency department physicians and nurses: multivariate logistic regression analysis.

	B	SE	aOR	95% CI	p-value
*Gender*					
Females^a^			1.0	---	
Males	1.014	0.420	2.76	1.21-6.28	0.017

*Smoking*					
No^a^			1.0	---	
Yes	2.732	0.397	15.37	7.06-33.45	<0.001

*Medication for sleep disorders *					
No^a^			1.0	---	
Yes	1.885	0.587	6.59	2.08-20.81	0.001

^a^Reference category, B: slope, SE: standard error, aOR: adjusted odds ratio, and CI: confidence interval.

Variables of on-call duties and intake of antianxiety medications were removed from the final logistic regression model (not significant).

## Data Availability

The data used to support the findings of this study are available from the corresponding author upon request.
